# Caring for relations and organizational success—conceptualization of an Augustinian leadership scale

**DOI:** 10.3389/fpsyg.2023.1170855

**Published:** 2023-06-26

**Authors:** Henri Slob, Paul van Geest, Harry Commandeur

**Affiliations:** ^1^Erasmus School of Philosophy, Erasmus University Rotterdam, Rotterdam, Netherlands; ^2^Erasmus Economics & Theology Institute, Erasmus Institute for Business Economics, Erasmus University Rotterdam, Rotterdam, Netherlands; ^3^Department of Biblical Sciences and Church History, Tilburg School of Catholic Thought, Tilburg University, Tilburg, Netherlands; ^4^Department of Applied Economics, Erasmus School of Economics, Erasmus University Rotterdam, Rotterdam, Netherlands

**Keywords:** Augustinian leadership, sense of belonging, affective commitment, morality, relational view, positive leadership, communal focus

## Abstract

While making great strides in recent decades to connect leadership and human flourishing, the positive leadership literature has yet to focus on the aspect of the communal. Based on a close reading of Augustine’s works, this paper examines Augustinian leadership and emphasizes the importance of a view on leadership that aims at community building and contains an ethical framework characterized by veracity. This leadership style is founded on *caritas* (Gr.: *agape*, Eng.: love) as the main motive for leaders. Based on Augustine’s thinking, this kind of love is defined as a way to attain knowledge. We identify four subconstructs to constitute an Augustinian leadership scale: Centrality of the community, Veracity, Empathy and Success (through temperance). We provide theoretical grounds for the distinctiveness of this leadership construct as compared with neighboring constructs. Finally, we propose a testable framework of Augustinian leadership with a direct effect on affective commitment as well as a mediated effect, and with a sense of belonging as the mediating variable. We provide ideas for future research and present practical implications of the theoretical insights on Augustinian leadership.

## Introduction

Leadership research has shifted from focusing on the leader to focusing more on the relationship between leaders and followers. Moreover, ethical considerations are increasingly being incorporated into more theories of leadership. The idea here is that greater attention not only to relationships in which human beings flourish but also to the broader organizational and ethical context will contribute to greater well-being for more people (Kelloway et al., 2013; Zhang and Song, 2020). However, these positive leadership theories still have a strongly individual focus, even though the follower is now the subject of interest rather than the leader (e.g., servant leadership, Van Dierendonck, 2011; Eva et al., 2019). However, in other cases, the leadership theory still focuses on leadership behavior or performance (e.g., transformational leadership, Wang et al., 2011). What is needed is a combined focus on the communal—that emphasizes the need to nurture a culture characterized by compassionate love—and an ethical framework characterized by veracity (*cf.* Nohria and Khurana, 2010; Rosette and Tost, 2010).

Positive leadership research has shown that organizational members are more willing to follow—and even go the extra mile for—leaders with high ethical standards and who value relationships. This manifests itself, for example, in high organizational citizenship behavior (Mo and Shi, 2017) or extra employee effort, both of which are related to higher performance by the firm (De Luque et al., 2008). In early Christianity, leadership, community and morality were very closely related, and even though the context of society and organization has changed, contemporary scholars frequently call for a closer connection to be made between those concepts once more (Sendjaya, 2005; Nohria and Khurana, 2010). What better place to search for answers than in the ancient source material itself, that is, going *ad fontes*, specifically in the texts of one of the church fathers who is widely considered to have greatly impacted Western thinking, namely Augustine (Pollmann and Otten, 2013). In the positive leadership literature we conceptualize Augustinian leadership as a leadership style connecting a communal perspective and morality, thus complementing the widely adopted insights from servant and transformational leadership. The ancient Christian thinker Augustine did not formulate a leadership theory himself, but through a close reading of crucial passages in his works in context we uncover his thoughts on leadership. By tapping into Augustine’s thinking, we are able to stretch our frame of reference and enrich our view on leadership.

A fundamental motive for human action and a prerequisite for human flourishing is “to belong” (Fromm, 1956). By engaging in reciprocal relationships, an individual develops an identity of their own (Buber, 1923/1978). Paving the way for this relational view of man, Scholastic theologians adopted a standpoint captured in the phrase: *homo est esse ad*. This means that a human *is*, because he is related to, as opposed to the Cartesian view that humans *are* because they think (*cogito ergo sum*). People base their self-conceptualization on, among other things, the groups they are members of and the leaders that appeal to their values and beliefs (Knez, 2016). The importance of a relational view of leadership has been pointed out by scholars developing the Leader–Member Exchange (LMX) theory of leadership (e.g., Graen and Uhl-Bien, 1995) and the social identity theory of leadership (Hogg, 2001; Steffens et al., 2021). Instead of focusing on the leader or the follower, the LMX theory incorporates both viewpoints together with the dyadic relationship. For this reason, surveys on LMX are designed to be taken by members as well as leaders, and concern behaviors of the leader combined with characteristics of the relationship [Graen and Uhl-Bien (1995), p. 237].

This paper addresses the conceptualization of an Augustinian leadership scale. First, the leadership dimensions are distilled by going *ad fontes* (i.e., reading Augustine’s texts). Next, a comparison is made with neighboring leadership constructs to identify overlapping and distinguishing components. Finally, a model is proposed with affective commitment as a possible result of Augustinian leadership, with a mediating variable. We thus contribute to the field of positive leadership by conceptualizing a leadership style with its effects, thereby providing a testable framework for future research.

## Augustinian leadership

Because Augustine’s thoughts on leadership are, as it were, implicitly and explicitly woven into treatises on themes such as community building, love or human happiness, we have selected as the starting point of our reflection on leadership those passages from his work in which this intertwining is most clearly expressed.

First, we provide insight into Augustine’s societal context, along with some biographical information. In the second part, the central work of Augustine (1961) is his *In epistolam Johannis ad Parthos*, which contains his reflections on the apostle John’s view on love. In the third part, we mainly draw on Augustine’s *De beata vita* and his *Praeceptum* (Augustine, 1967, 1970). These works contain practical guidance for, respectively, living a virtuous life and leading a community. In his *De Civitate Dei*, Chapter 24, Augustine defines a community as “an assemblage of reasonable beings bound together by a common agreement as to the objects of their love.” Here again, love is the central construct to understanding a community and its members. Because of the centrality of this construct, we first discuss its layers of meaning.

In the following, we present Augustine’s thoughts insofar as they present insights into leadership from a business economic and psychological viewpoint. We thus do not aim to present the full spectrum of Augustine’s thoughts, and this will mean losing some of the richness of his works. However, we do provide a coherent leadership framework that is based on scholarly insights into Augustine’s teaching. All insights below have previously been published in theological, peer-reviewed journals.

## Context

Few thinkers can rival Augustine’s (354–430) influence on Western anthropology, theology and cosmology (Pollmann and Otten, 2013). His career as a teacher of rhetoric was made in Madaura, Carthage, Rome and Milan. After his baptism in Milan in 387 he developed into an extraordinarily prolific writer. As bishop of Hippo—he was ordained in 395—he wrote a great number of sermons, letters, biblical commentaries and longer works in which he emphasised the primacy of grace, arguing that this preceded good will. He also composed treatises in which he attempted to safeguard the unity of the church, for instance by accusing the Donatists of seriously wounding the church, the Body of Christ, through their schism, as we shall see below. His examination of conscience and the self-analysis performed in his *Confessiones*, as well as his account of history and of the ideal social and societal order in *De civitate Dei*, composed to prove the value of Christianity, have been most influential throughout the centuries. But at the end of his life, Augustine had to leave four works unfinished. One of these was his *Retractationes* (426–427): the catalogue of his works in chronological order, each accompanied by criticisms, corrections, and comments. It was intended as a toolbox for the expansion and spread of Latin Christendom (Drecoll, 2002).

In the decades before he was born, Africa had experienced an unprecedented economic boom, having developed into the granary of the late Roman Empire. The region where Augustine grew up was very prosperous in the third to early fourth centuries, which was reflected in the construction of public monuments, such as amphitheaters, in the cities. But in the fourth century, prosperity waned in the cities of what is now North Africa. The region in which Thagaste was located became more agrarian again and famine was not uncommon in the countryside. Born the son of an admittedly impoverished but still Roman aristocrat, Augustine, however, did not really know poverty. Even as a teacher and later as a bishop, he belonged to an elite. It was during his period as a bishop that he began devoting thoughts to leadership. He did so at a time when leadership was not upheld democratically; not infrequently, the law of the strongest applied.

## Love as the key motive for human behavior

Central to Augustine’s thinking on human relations is *caritas* (Eng.: love) as a way of achieving knowledge (Lat.: via *amoris*). In Augustine’s thinking, the substantive “love” has many meanings. The layers of meaning he attributes to the word are sometimes not even separable from each other. In his *In epistolam Johannis ad Parthos* (*ep. Io. tr.*, 415/1961), for example, it turns out to be a very layered concept. In its highest form, love is God himself (415/1961, 1.11; 8.5–8.7). However, love also appears to be a force in man because it is supposed to be a commandment and the ultimate goal of all commandments (415/1961, 1.9; 5.2; 6.4; 10.4; 10.5). By “love” is expressed a fundamental attitude and way of life (415/1961, 5.2; 9.1). It also stands for an inner force that, like desire, creates in man a receptivity towards God (415/1961, 6.8; 6.10; 6.12). As an inner force, moreover, love appears almost inseparable from love as a gift of grace (*ep. Io. tr*. 6.8). Love cannot exist without the Spirit of God. But it simultaneously takes shape in personal love for one’s neighbor (415/1961, *6.10; 7.5; 7.6*. *cf.* 1 John 4: 7–8).

In his *In epistolam Johannis ad Parthos*, Augustine thereby also indicates the different stages to be distinguished in the growth of love. For example, he relates the physical love of married couples, which is related to the creative urge that is eros, to God by referring to it as a first stage in the development towards real Love. He also sees the willingness to do something for another and compassion for someone in need as the first stage in this development (415/1961, *5.12*). This love should be further nourished by the word of God. When this happens, according to Augustine, the motive (“love”) and the basic attitude (“humility”) coincide. He sees the willingness to give one’s life for a fellow human being, or even for an enemy, as a sign of perfect love. It is in this, that man still senses the unknowable being of God (415/1961, *5.12; 6.1; 6.13*).

Of crucial importance in Augustine’s view of *caritas* is that love involves a way of knowing. The via *amoris* is a way in which the creative urge (*eros*) is embedded in *agape* (*caritas*). Since Descartes and the Enlightenment, we have seen reason and reason alone as a capacity by which we know and arrive at substantiated insights. For Augustine, the capacity to love is crucial in order to arrive at knowledge, insight and wisdom. In the second book of *De doctrina christiana* (397/1866), Augustine writes that the insight into the insufficiency of the faculty of knowing can be overcome by love as a way of knowing, because it eliminates pride (Augustine, 1866). Moreover, Augustine maintained that humility is the seedbed of love and leads to deepened insight into the self, the other, the mystery of life. Loving someone would even lead to the highest possible form of knowledge (Van Geest, 2011).

In contrast with the first categories of love, *eros* and *philia*, this latter conceptualization of *agape* (or *caritas*) is not primarily concerned with admiration for the other, nor is it a form of contemplation; rather, it is an active, moral form of loving the other (Van Geest, 2011, p. 172; see also Levinas, 1994). This love directed to the other is based on the dignity they possess as a human being instead of a dignity resulting from either their societal status, possessions or capabilities.

**Figure 1:**
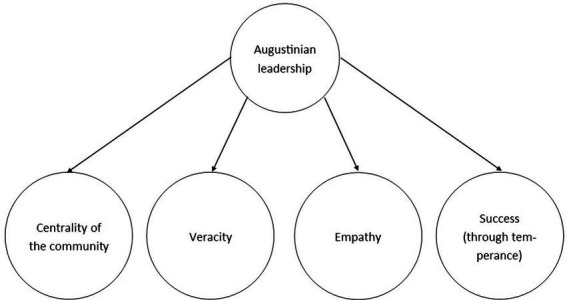
Augustinian leadership and its subconstructs.

The kind of love to which Augustine frequently referred was thus not of the romantic or erotic kind; rather, he meant to point out the importance of *compassionate* or *neighborly love*. For him, the power of *caritas* remains a mystery, just as the source of love (God) is. In *epistolam Johannis ad Parthos*, he states that no one can say what face, what form, what stature, what feet or what hands love has. But Augustine hastens to add that love has hands, for they reach out to the poor; eyes, for they see who is in need (415/1961, 7.10). In the context of this concept of love, he develops in his works four key leadership constructs that cannot be understood without keeping in mind their purpose: to love (see Figure 1). These four are: Love for the community, love for truth, love for the individual and ensuring durability for the community. We label these aspects respectively: Centrality of the community, Veracity, Empathy and Success (through temperance). We now turn to elaborate on each aspect of Augustinian leadership.

## The dimensions of Augustinian leadership

As stated before, Augustine did not form a comprehensive leadership theory. He was, however, one of the first to systematically reflect on human behavior, the will and memory. In doing so, he countered ideas of the Platonic school that praised the rational capacities of human beings and thought honor to be a laudable cause to strive for. Augustine thought humility should be the basic attitude for leaders and love the key motive for behavior. People’s will and the *ordo* which they live in is strongly determined by factors outside people’s influence. Possessions and capabilities are granted to people by *gratia* (Eng.: grace). In the era he lived in, this anti-meritocratic view was strongly against the view of man, represented by his opponent Pelagius, who placed greater emphasis on the fact that people determine their own future. Certainly, in his later period, Augustine commented on this, maintaining that man’s recognition that he owed his talents, educational opportunities and development mainly to others and to the Creator would make him more humble (Drecoll, 2020). Centuries later, influential thinkers like Thomas Aquinas and John Calvin, built on Augustine’s thinking, making him a foundational thinker for their view on subjects such as love and leadership, among other things.

In Augustine’s thoughts on man and human existence, the assumption is not that man *is* because he thinks (Descartes), even though he presumes reason to be man’s highest capacity. In his anthropology, the human being is first and foremost a relational being. The human being *is* because he is able to establish relationships with others. This implies dualities under which leaders find themselves placed. Should a leader be result-oriented or also keen to impress upon employees that their work has meaning? Should they be action-oriented or condition-oriented, as Augustine envisaged when he developed a leadership model in his *Praeceptum*? In the Augustinian leadership model, the guiding questions posed by the church father to his people read, namely: what do you need; what preconditions may I create for you so that you not only experience yourself as a meaningful link in a community, but also remain focused on the common goal of us together; a goal characterized by the pursuit of a good relationship and reciprocity? (*cf. Praeceptum* 1; 5).

### Centrality of the community

The Augustinian leader prioritizes a flourishing community over self-interest and pure altruism. Clarifying this prioritization, Augustine in his *Praeceptum* postulates a reciprocal relationship between the well-being of the community and of each individual participating in it, as well as between the *praepositus* (leader, overseer) and the community’s members (Van Bavel, 1959; Verheijen, 1980). Every member of the community, including the leader, needs the others to flourish (*Praeceptum* 1,3; *De Civitate Dei XIX*). Together they are travelling through life, helping each other in their development. One of the conditions for a community to flourish is that participants cannot place their self-interest above the communal interest. Moreover, the community is not supposed to be a vehicle for self-actualization nor to consolidate their power. Augustine tells his confreres in the fifth chapter of his *Praeceptum* that they must never work to pursue their own interests but should be all the more zealous about their work because it benefits the community (397/1967, 5,2). Therefore, he opens his *Praeceptum* with the admonishment to live together in unity, “with one soul and one heart, towards God.” (397/1967, *Praeceptum* 1; *cf.* Acts 4: 31–35). Just as in the earlier *Ordo Monasterii*, in the *Praeceptum* he identifies this aspect as the path to God. At the end of the first chapter, he associates the path to God (*in Deum*), which is established through unity of heart and soul, with the way we treat our neighbors (397/1967, *Praeceptum* 1; Van Bavel, 1996). With this inclusion in the first chapter, Augustine indicates that the perfection of the individual is related to the wholeness of the community and to the way we relate to others. And yet this great unity of heart and soul is supported mainly by the way in which people live together in everyday life: within the inclusion (Van Geest, 2020).

In order for the community to have a long lifespan, the Augustinian leader will strive for *unitas* (Eng.: unity), which presupposes compassionate *disciplina* (Eng.: discipline or correction) (Van Bavel, 1959). The latter is merely a method, never a goal in itself. To employ discipline in a predictable and just way, the Augustinian leader defines the community’s boundary conditions. This way, guidance is offered to the members and appropriate measures can be taken when someone does not conform to the agreements made (Van Bavel, 1959). Augustinian leadership thus facilitates *concordia* (Eng.: harmony).

### Veracity

Next, to *unitas*, the Augustinian leader sees veracity as the central component of morality. This means that speaking the truth is paramount in the ethical considerations of this leader. Lies, defined as willingly telling anything other than the truth or as withholding the truth, are not allowed (Van Geest, 2007). This in contrast to other church fathers who found white lies justifiable because, for example, it would not show mercy for a dying mother to be told in the hour of her death that her son had died in war. Although Augustine recognizes that he would not meet his own standard in such a situation, he still argues that the mother should be told the truth. He explains his point by stating that if people do not speak the truth, they do not stay on the track of the Truth that encompasses the world (Van Geest, 2017). This is because of the deteriorating effect lying has on the relationship: it makes group members doubt the trustworthiness of the person who is lying. In any case of defective behavior, be it lying or something else, the Augustinian leader enacts discipline compassionately. This means that a dialogic approach is chosen, instead of measures of hard power to instill fear in the person who wronged the group. This individual should be pointed toward the desirable behavior, and the leader’s efforts should be aimed at maintaining this person as a member of the group.

### Empathy

Here, we clearly see the importance of empathy in Augustinian leadership. The Augustinian leader grants each member time to become accustomed to the group norms and disciplines mercifully in the case where a member does not live up to these norms (*Praeceptum* 7; Schrama, 1991; Burt, 1999). The reason for this being that the norms are neither ends to strive for, nor a way to measure each members’ perfection. Norms are in place to facilitate the development of each individual towards a better way of dealing with others (Köpf, 2007). This process culminates in *concordia* (Eng.: harmony). Moreover, while the leader is not primarily tasked with every member’s self-actualization, it is part of their responsibility to acknowledge every individual’s singularity and to adapt the way in which each group member is treated, according to their needs. This means that the strongest members are being challenged enough to develop personally, while the weaker members receive extra support to accomplish the given tasks. In the Augustinian leadership model, the church father aims at providing the preconditions needed for his people to thrive and experience themselves as a meaningful link in a community which strives after good relationships and reciprocity (*cf.* Augustine, 397/1967, *Praeceptum* 1,5; Zumkeller, 1968).

There is also a tension between leadership that focuses on thinking and doing and leadership that finds identity and development of primary importance. In the Augustinian leadership model, these two forms of leadership coincide. A bishop, *sermo* 340 shows, is good not only because he has worked out a good strategy for the future of his local church or shows himself to be a skilled administrator who keeps the finances well organized. Above all, a good bishop also knows himself anchored in the community he has to lead. His function and responsibilities do not separate him from his people. Feeling part of the community is the basis for good leadership. This means the leader should not place himself above the community: that is, he has to be humble. Indeed, he can do well only when he is able to experience the power of consolation and encouragement. Thus, unity is not only posited as essential for the community, but also within the leader, who should be *integer*: *praxis* and intentions should be one (*sermo* 340a).

### Success through temperance

In order to maintain the community and provide stability for the group members, the Augustinian leader strives for success, albeit through temperance. To him, success is equal to the durability of the community and to attain this, temperance is the required attitude (*Praeceptum* 1,3). The Stoic *ne quid nimis* principle (“nothing in excess”) is central to Augustine’s thought on this matter (Lawless, 1987). Too little or too much of anything is not desirable. He writes: “Not that he [the superior] must give everyone an equal share, because you are not all of equal strength, but he must give to each one what he personally needs.” Thus, in *Praeceptum* 1,2 (397/1967), he translates the Stoic *ne quid nimis* principle into specific guidelines (Lawless, 1987; Van Geest, 2020). To illustrate his point, Augustine uses the example of Sergius Orata, a wealthy entrepreneur and inventor in the Roman Empire (n.d./1970). This man owned luxurious spas and was astute. However, due to his perceptiveness, he knew all too well that all his possessions could be lost due to some adversity. Augustine’s conclusion in his *De beata vita* was that this fear of losing his possessions kept him from achieving true happiness. The same is true, however, for the poor whose worry stems from the lack they experience. In order to achieve true happiness, one should have just enough of everything in order not to worry about providing for one’s own community while also not living in fear of losing the acquired wealth. To know when “wealth sufficiency”—that is, success—is achieved, wisdom (Lat.: *prudentia*) is needed. A wise and successful person is one who knows how to live with temperance.

Furthermore, Augustine states that *temperantia* is not a virtue solely applicable to the amount of personal or communal wealth. He also connects this concept to living a balanced life (Lawless, 1987; n.d./1970, IV.25). Augustine identifies seven—mutually interacting—"layers” constituting a balanced life (n.d./1970, IV.25; Van Geest, 2004). First, no life exists without body and breath. The second layer consists of the senses, e.g., seeing, feeling, and tasting. The intellectual abilities, as well as manual and artistic competencies, of human beings form the third part. The fourth aspect of the balanced life is morality. The final three layers form the transcendent part of human life, with the soul becoming one with God as the final layer. Modesty, or temperance, is essential for development of the soul and for living a balanced life and should be visible through humility. Augustine even states that a leader should be cured from *superbia* (“pride”), and this would be seen to be accomplished when the person becomes humble. In the fourth chapter of his *Praeceptum* (397/1967), Augustine begins by pointing out to his confreres that they must not try to stand out by their clothes, but by their attitude to life. The reason for that is that only a humble person can relate to others in a way that helps them grow and live an ordered life in contentment.

## Neighboring leadership concepts

The Augustinian view on leadership contributes to the understanding of leadership centered on the organization as a community and containing moral dimensions. In this field, several leadership constructs already exist. In our comparison with neighboring constructs, the focus will be on servant leadership, transformational leadership and LMX. This is because these theories have generated a great deal of interest in leadership research and they either contain a moral component (servant and transformational leadership) or have a relational focus (LMX). For that reason, we do not consider other leadership theories, such as authentic leadership, where the main focus is on the authenticity of the leader. Augustinian leadership, we argue, can be differentiated from these leadership styles. The summary of our discussion can be found in Table 1.

**Table 1:** Comparison of Augustinian leadership with neighboring leadership constructs.

	Augustinian leadership	Servant leadership	Transformational leadership	Leader-member exchange theory of leadership
Nature of theory	Normative	Normative	Normative	Descriptive
Role of leader	To create a lasting, successful community	To serve followers	To inspire followers to pursue organizational goals	To develop positive relationships with followers
Role of follower	To serve the communal goals	To become wiser, freer, more autonomous	To pursue organizational goals	To develop positive relationships with leaders
Moral component	Explicit	Explicit	Unspecified	Unspecified
Moral contents	Veracity, moral development	Moral development	Unspecified	Unspecified
Outcomes expected	Affective commitment, employee well-being, organizational success and endurance	Follower satisfaction, and commitment to service, societal betterment	Goal congruence, increased effort, satisfaction, and productivity, organizational gain	High LMX-satisfaction, mutual trust, increased effort
Individual level	Desire to build community	Desire to serve	Desire to lead	Desire to relate
Interpersonal level	Leader empathically helps follower develop	Leader serves follower	Leader inspires follower	Leader exchanges with follower
Group level	Leader creates boundary conditions within which members act autonomously	Leader serves group to serve members’ needs	Leader unites group to pursue group goals	Leader develops different exchanges with each person
Organizational level	Leader strives for satisfactory profit to maintain the community	Leader prepares organization to serve community	Leader unites followers to pursue organizational goals	Unspecified
Societal level	Leader guides society through dialogue and justice	Leader leaves a positive legacy for the betterment of society	Leader inspires nation or society to pursue articulated goals	Unspecified

Adapted from Barbuto and Wheeler (2006).

While servant leadership focuses on the individual, the proposed Augustinian view on leadership prioritizes building a community. Community building has been considered a vital ingredient for servant leadership (Laub, 1999), but Barbuto and Wheeler (2006) conclude that this characteristic does not belong in the Servant Leadership Questionnaire since it is not unique to this leadership style. Moreover, in the systematic review on servant leadership by Eva et al. (2019) only one of the three recommended measures of servant leadership behavior contains items on the communal focus of the leader. In this measure, the SL-7 compiled by Liden et al. (2015), the community consists of the organization’s surroundings. Adding to this, Yukl (2010) states that servant leaders prioritize social responsibility over short-term performance of the organization. While this may seem laudable, it also makes it more difficult for the leader to resolve the conflicting needs of team members and the organization (p. 421). In Augustinian thinking, this problem is less likely to arise because the leader is not called to serve in the first place—although serving is an important aspect of Augustinian leadership (Shirin, 2014). However, the Augustinian leader is expected to exercise authority when the situation calls for it.

The fact that Augustinian leadership gives priority to building a community also makes it possible to distinguish this leadership style as a complement to transformational leadership. The latter is mainly oriented toward idealized influence, or charisma (that is, at the person and behavior of the leader) (Bass, 1999; Yukl, 2010; Van Knippenberg and Sitkin, 2013). Through this behavior, characterized by idealized influence and intellectual stimulation, the leader is expected to align the personal interests of followers with the interests of the organization (Bass, 1999). Transformational and Augustinian leadership share some resemblance on this point, because followers should be oriented to communal interests above self-interest. However, all members of the organization going in the same direction is not the same thing as forming a community. In the proposed model of Augustinian leadership, a reciprocal relationship between individual flourishing and serving communal goals is a core ingredient of forming a closely knit community. Moreover, in order to achieve alignment of interests, the transformational leader starts with a vision and by articulating this he engenders respect and loyalty in the followers (Banks et al., 2016). Thus, the leader and his actions are the primary focus of this leadership style, which distinguishes this style from Augustinian leadership, since the proposed leadership framework emphasizes the importance of the community over the individual importance of either the leader or the follower. Finally, the communal focus of the Augustinian leader distinguishes the style from LMX theory, because of the dyadic focus of the latter. Here, collectivity is defined as the aggregation of dyads (Graen and Uhl-Bien, 1995). Within these collectivities, the network of relationships which forms the leadership structure is mapped onto the task structure of the organization, analyzing relationship effectiveness. This shows the dyadic and task-oriented focus of LMX theory. It leaves out aspects of culture and the sense of belonging to a group that serve to bind the dyadic relationships together.

The Augustinian leader acknowledges the importance of communal success in the long run to provide for and maintain the community. As stated above, leaders and members are called to place communal goals above self-interest. By doing so, they ensure that they will receive what they need when the fruit of their work is distributed among the community’s members. Yukl (2010) stated that in servant leadership the prioritization works the other way around, that is, the employee’s interest is put at the top. This makes it more difficult for a true servant leader to make decisions that would benefit the organization in the short run while benefitting its members as well—for example, by securing employment opportunities through obtaining higher financial buffers. Theory on transformational leadership does take organizational gain into account and is aimed at inspiring followers to transcend their self-interest (Bass, 1999). Hence, this theory shows some resemblance to Augustinian leadership, though the latter explicitly strives for success through temperance and within an ethical framework characterized by veracity. This prescriptive ethical framework differs from the moral basis of transformational leadership because, in the latter theory, identifying the content of morality is left to respondents rating their leader (Bass and Steidlmeier, 1999; Hannah et al., 2014; Hoch et al., 2018).

Its emphasis on the importance and content of morality, via veracity, is expected to distinguish the Augustinian leadership style from servant and transformational leadership, because morality in these latter theories means the moral development of the follower (Bass, 1999; Barbuto and Wheeler, 2006). Moreover, with regard to servant leadership the moral component is not clearly conceptualized at all (Lemoine et al., 2019; Nullens, 2019). Leaders are expected to elevate followers’ moral consciousness and their capacity to act ethically, either through exemplary behavior, e.g., *idealized behavioral influence*, or by sharing (personal) values and beliefs (Bass, 1999). According to Yukl (2010), transformational theory is a rather behavior-oriented leadership style, while servant leadership, belonging to the stream of ethical leadership, emphasizes morality and values more strongly than transformational leadership (see also: Lemoine, 2015; Hoch et al., 2018). That is why the row headed “Moral component” in Table 1 states that this aspect is “unspecified” for transformational leadership. In LMX theory, morality is never mentioned as an aspect of relationship quality, which is the main variable in this field (Mahsud et al., 2010).

Lastly, empathy is considered a distinctive aspect of Augustinian leadership compared with servant leadership, transformational leadership and LMX. In *Praeceptum 5* (Augustine, 397/1967), Augustine asks the follower and leader to open themselves to each other in order to be able to empathize with the other’s person and situation. While empathy is considered to be an integral part of servant leadership by some scholars (Spears, 2010), Barbuto and Wheeler (2006) state that this construct, along with listening, is “not unique to servant leadership” (p. 319). Their conclusion after composing and testing the Servant Leadership Questionnaire is that the items associated with empathy should be dropped. Empathy, as operationalized here, partly overlaps with the construct *individualized consideration,* which is an element of transformational leadership (Bass, 1999). However, this construct mainly focuses on the opportunity of personal and professional growth, comparable with the construct *growth* in servant leadership (Barbuto and Wheeler, 2006), and does not take into account the importance of emotions and perspective-taking (Avolio and Bass, 1995, p. 202). Finally, empathy as a core part of Augustinian leadership clearly distinguishes this theory from LMX, because in the latter this construct is not considered to be fundamental (Graen and Uhl-Bien, 1995). Rather, taking listening as a proxy for empathy, studies have found this to be an antecedent to high quality relationships, that is, high LMX (Mahsud et al., 2010; Lloyd et al., 2017).

The overall comparison between Augustinian leadership and the three neighboring styles of leadership is provided in Table 1. This table was first composed by Barbuto and Wheeler (2006) and adapted for the purposes of this paper. As they did not explicate the contents of morality, we added the row headed “*Moral contents.*”

## Augustinian leadership, sense of belonging and affective commitment

**Figure 2:**
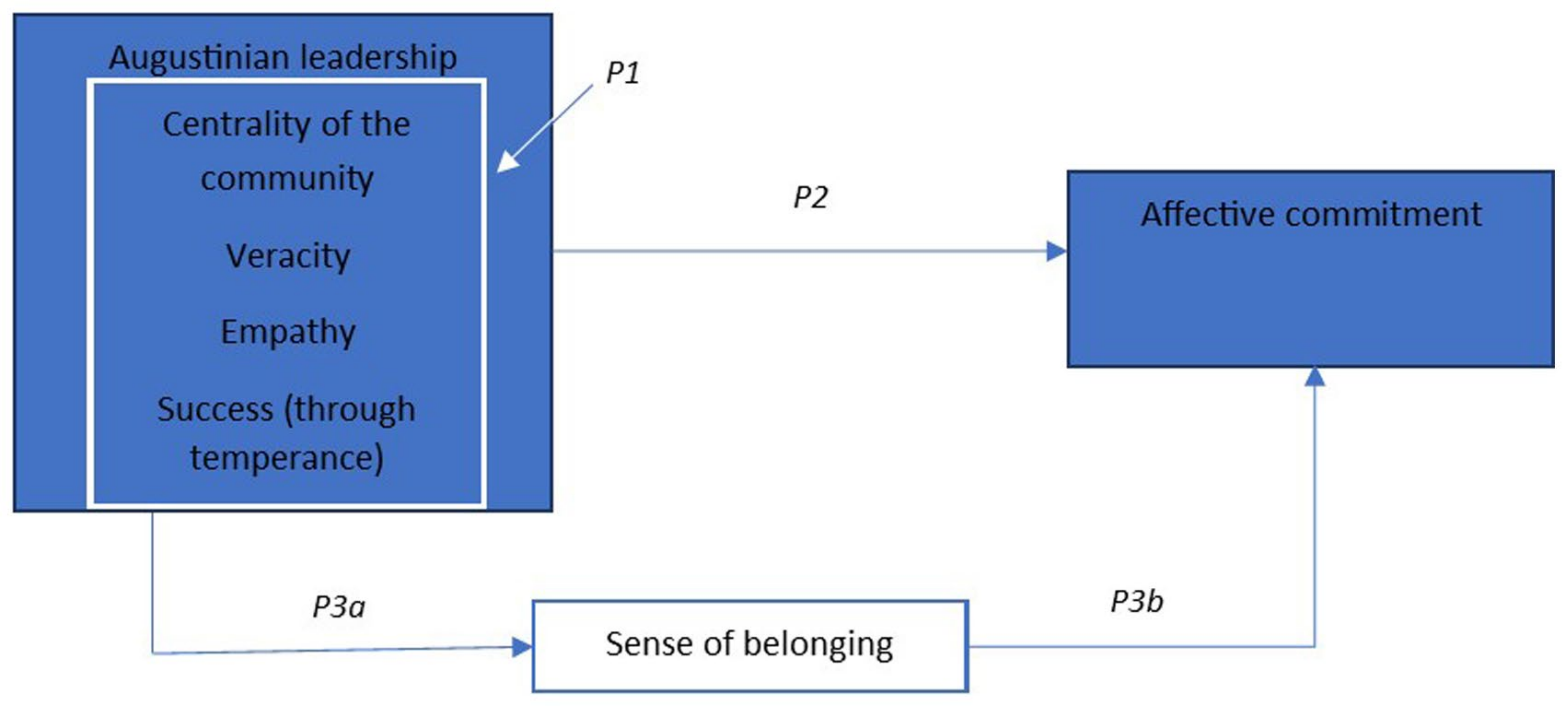
Proposed relationship between Augustinian leadership, sense of belonging, and affective commitment.

Besides conceptualizing Augustinian leadership based on what it is, we aim with this paper to provide insight into the expected outcome of applying the principles of Augustinian leadership, including a mediating effect (see Figure 2, *cf.* Carasco-Saul et al., 2015). This forms a starting point for identifying the practical relevance of Augustinian leadership, with possibilities for future research to crystallize our understanding of this leadership construct and its antecedents, correlates and outcomes. For this first framework, we employ the literature on organizational commitment since this deals with the attachment of individual interests to social systems (Kanter, 1968). These systems, or communities, play a significant role in Augustinian teachings (Augustine, 397/1967). Moreover, organizational commitment has proven to be a highly relevant construct in explaining employee well-being, turnover intention and job satisfaction (Wiener et al., 1987; Mathieu and Zajac, 1990; Panaccio and Vandenberghe, 2009).

Below, we discuss our proposed conceptual framework on Augustinian leadership in relation to the extant literature. Per relationship within the model, we form a proposition which follows from the theoretical discussion.

Figure 1 illustrates our model of Augustinian leadership. We identify the four dimensions by going *ad fontes*: Centrality of the community, Veracity, Empathy and Success (through temperance). Consistent with the development of other leadership scales, these four dimensions are expected to correlate strongly and function as indicators of Augustinian leadership as a latent construct (*cf.* Dahling et al., 2009). Following this, our first proposition is:

Augustinian leadership is expected to consist of four dimensions, namely: communal focus, veracity, empathy, and success (through temperance).

Converging the work on organizational commitment and developing the Organizational Commitment Questionnaire (OCQ), Mowday et al. (1979) laid the foundation for developing further insights into this concept. In their seminal paper they define organizational commitment as “the relative strength of an individual’s identification with and involvement in a particular organization” (p. 4). Although this is a multifaceted definition, it is still one definition for a layered construct. Currently, scholars agree that organizational commitment is a multidimensional construct and identify three forms of commitment: affective commitment, normative commitment and continuance commitment (Meyer and Allen, 1991; Meyer et al., 2002). We will first discuss the latter two briefly, and, since *affective commitment* is chosen as the main construct, we will dive into this construct more deeply later. Normative commitment is based on an individual’s belief that he “ought to” stay, a belief that stems from internalized norms leading to the conclusion that staying with the organization is a moral obligation (Allen and Meyer, 1990). Continuance commitment is grounded on the calculative approach employees take to deciding whether to stay or leave. When leaving the organization is accompanied with (perceived) higher costs than staying, an individual will stay, and vice versa (Becker, 1960). Finally, affective commitment measures whether an individual “wants to” stay at the organization, based on emotional attachment to, identification with and involvement in the organization (Meyer and Allen, 1991). Based on this characterization of affective commitment, it is clear that the emotional aspect is complemented with an evaluative aspect. In Meyer and Allen’s (1991), piece the evaluative part of affective commitment can be seen in, among other things, the importance of the fulfillment of pre-entry expectations (pp. 70 and 75). The antecedents for this form of commitment, identified by (Meyer and Allen, 1991; see also Mathieu and Zajac, 1990), are “personal characteristics,” “organizational structure” and “work experiences.” This last category consists of objective and subjective job characteristics, such as autonomy, organizational support and supervisor consideration (p. 71). DeCotiis and Summers (1987) identify a similar antecedent to organizational commitment, calling it “organizational processes.” They state that these processes “are the way things get done in an organization” (p. 451), including leadership. Ethical leadership, with ethical climate as the mediating variable, has indeed been shown to impact affective commitment (Demirtas and Akdogan, 2015). Kim (2014) found a direct and indirect, via clan culture, effect of transformational leadership on affective commitment. Hence, because Augustinian leaders prioritize building and maintaining the community with individual consideration, we expect members of the organization to be more likely to want to stay at the organization, that is, to show affective commitment. Following this, our second proposition is:

Augustinian leadership is expected to have a positive effect on affective commitment.

The communal focus of Augustine’s thinking is not merely a postulate with conclusions on the macro-level. In his writings, he states that every human being always belongs to some group, and at the macro level to the human species. Belonging is a disposition for every person, and a gift (Augustine, 397/1967, Praeceptum 1 and 5). At the same time, the leader is called to actualize the sense of belonging (Augustine, 397/1967, Praeceptum 5). This construct is relatively new in leadership studies. Most studies related to it are conducted in educational research (e.g., Freeman et al., 2007; Allen et al., 2018). For the development of the instrument that is the sense of belonging, Hagerty and Patusky (1995) tested their scale with students, patients and Roman Catholic nuns—all individuals associated with the public sector. However, the closely related concept of organizational identification has gained a lot of scholarly attention. If a member strongly perceives himself to be part of the organization, organizational affairs become psychologically attached to the individual’s perceived success (Ashforth and Mael, 1989). This process is called organizational identification and this belongs to the stream of research into social identification (Ashforth et al., 2008). Tajfel (1982), in his foundational paper on the topic of social identity theory, states that for identification two components are necessary, namely: a cognitive component that concerns “awareness of membership” and an evaluative component that means this awareness is accompanied with “some value connotations” (p. 2). A study by Cameron (2004) shows that a three-component model would be even stronger. His model consists of “Centrality,” the “In-group affect” and “In-group ties.” The first concept refers to the idea that not every involvement in a group is as central to an individual’s self-categorization as other group memberships. Second, the in-group affect is closely related to Tajfel’s evaluative component of identification, though here the emphasis is on the emotional value of group membership. The third aspect, in-group ties, is closely related to sense of belonging, because this aspect is operationalized as the extent to which individuals feel they are part of the group (Cameron, 2004, p. 243). Harris and Cameron (2005) argue that this multi-dimensional approach to identification is needed in order to explore more detailed relationships between organizational identification and its consequences. For example, in their study they find that the more emotionally relevant dimensions of identification are more strongly correlated to self-efficacy. For the purposes of this study, we focus on sense of belonging as an aspect of organizational identification.

Organizational identification has been connected to organizational commitment as an antecedent and an overlapping construct (Ashforth and Mael, 1989; Wiesenfeld et al., 2001; Riketta, 2005). It has been shown to positively impact employee performance and creativeness (Riketta, 2005; Hirst et al., 2009). Organizational factors and interpersonal factors, specifically leadership, have been identified as antecedents of organizational identification (He and Brown, 2013; Luo et al., 2022; Niu et al., 2022). For example, transactional and transformational leadership appear to have a positive impact on identification, moderated by positive and negative affectivity (Epitropaki and Martin, 2005). Based on the foregoing discussion, we expect a positive, direct relationship between Augustinian leadership and sense of belonging. Following this, our third proposition, part a, is:

Augustinian leadership is expected to have a positive effect on sense of belonging.

Meyer and Herscovitch (2001) proposed a general model of workplace commitment with the bases of the different kinds of commitment and the connection with behavior. For this, they adopt the three-component model of organizational commitment of Meyer and Allen (1991). They identify identity and personal involvement as bases for affective commitment (Meyer and Herscovitch, 2001, p. 317). Based on this model, we expect a positive effect of sense of belonging, as a dimension of organizational identification, on affective commitment. Though these concepts have been used as synonyms, currently the consensus is that they are indeed two separate constructs (Mael and Tetrick, 1992; Riketta, 2005). The difference has been captured by stating that organizational identification is the process and commitment is the output (Meyer et al., 2004; Dávila and García, 2012). Ashforth and Mael (1989), influential scholars on this topic, state in their paper that identification is organization-specific: that is, an individual identifies with a particular organization. Commitment to organizational values can be transferred to another organization without personal, psychological costs. However, organizational identification leads to an individual feeling psychologically connected to the organization, that is, feeling as one, thus raising psychological costs if the member were to leave the organization (Van Knippenberg and Sleebos, 2006). The reason for this has to do with identification being self-referential while commitment is exchange-based (Van Knippenberg and Sleebos, 2006, p. 579). Because of this difference, Van Knippenberg and Sleebos conclude that social exchange analyses should incorporate not only the exchange component, commitment, but also concepts of self-definition, such as organizational identification.

Several studies have found a positive effect of organizational identification on affective commitment. For example, Bergami and Bagozzi (2000) have shown that cognitive organizational identification leads to affective commitment, with the latter defined specifically as emotional attachment. In their analysis, they tested a reciprocal relationship between identification and commitment but did not find an effect of commitment on identification (p. 570). Others find the constructs to be distinctive, although they show some overlap (e.g., Edwards, 2005; Van Knippenberg and Sleebos, 2006). Organizational identification is shown to correlate more strongly with extra-role behavior and job involvement, while (affective) organizational commitment is stronger correlated to job satisfaction and intent to stay (Riketta, 2005). Concluding, we expect sense of belonging, as a dimension of organizational identification, to positively affect affective commitment. Following this, our third proposition, part b, is:

Sense of belonging is expected to have a positive effect on affective commitment.

## Conclusion and suggestions for future research

Leaving the focus on power and on the individual displayed by other leadership theories, we propose Augustinian leadership as a leadership style that answers the call for more communally focused and morally laden leadership constructs. This style of leadership is theoretically distinct from neighboring leadership constructs such as servant leadership and transformational leadership. Moreover, we provided a testable framework with propositions, thus providing clarity about the potential practical relevance of Augustinian leadership. The next step would be to test this framework and statistically assess the distinctiveness of Augustinian leadership compared to closely related theories of leadership.

The first edition of the Augustinian leadership scale has been developed and a face validity check with experts has been performed (Boateng et al., 2018). This scale was tested in a pre-study with 399 respondents recruited via Prolific, a platform recommended by Eyal et al. (2021). We are currently in the phase of evaluating the survey items. Through this process we will identify the items needed in the following phase of scale development and items that should be added or dropped. When this has been done, we will administer the scale on a similarly sized sample (Churchill, 1979). On this data we will perform an exploratory factor analysis to determine whether the proposed model of Augustinian leadership, with four dimensions, is present in the data (Boateng et al., 2018).

For this first conceptualization of Augustinian leadership, we identified four subconstructs. Future research into this leadership construct could dive deeper into *humilitas* and *auctoritas* as concepts influencing Augustine’s view on leadership and leader behaviors.In addition to the proposed conceptual framework, future work on Augustinian leadership could look into other positive organizational outcomes—for example, extra-role behavior or turnover intention. Besides affective commitment, other positive outcomes correlated with Augustinian leadership could be employee well-being, with affective commitment as mediating variable (Meyer et al., 2002), and flourishing in the midst of turbulence (Urick et al., 2021). This leadership style could also benefit from studies looking into its contingencies. For example, individuals’ personalities or the industry context might affect the interaction between Augustinian leadership and expected positive outcomes (*cf.* Epitropaki and Martin, 2005).

## Practical implications

As a form of positive leadership, Augustinian leadership could help us better understand which personal strengths and characteristics would be favorable when selecting leaders within organizations. With its emphasis on creating a community with clear moral boundaries (that is, in turn, fertile ground for positive work outcomes), Augustinian leadership would be advantageous for organizations as well as individuals to tap into Augustine’s thinking and the lessons we can learn from him today.

Given the meritorious nature of this leadership style inside the organization, it could also serve as a construct to be taught in business administration and executive education. This would offer students a more diverse view on leadership, adding the communal and morally laden approach to the transactional and power-focused styles of leadership. With its explicit moral component and an ethical framework characterized by veracity, Augustinian leadership could also prove useful as a complement to transformational leadership, which is a style already well received in consultancy and executive education.

Finally, because the Augustinian leader combines an empathic approach with guidance for the community via a clear ethical framework, this leadership construct is likely to be useful in the modern world, which is facing a great deal of turbulence. Understanding and attending to the individual needs of members of the community while also providing clarity on what is “right” are expectedly ingredients in maintaining hope for a better, more stable future.

## Data availability statement

The original contributions presented in the study are included in the article/supplementary material, further inquiries can be directed to the corresponding author.

## Author contributions

HS, PG, and HC contributed to the conception of this study. HS wrote the first draft of the manuscript. PG wrote sections of the manuscript. All authors contributed to the article and approved the submitted version.

## Conflict of interest

The authors declare that the research was conducted in the absence of any commercial or financial relationships that could be construed as a potential conflict of interest.

## Publisher’s note

All claims expressed in this article are solely those of the authors and do not necessarily represent those of their affiliated organizations, or those of the publisher, the editors and the reviewers. Any product that may be evaluated in this article, or claim that may be made by its manufacturer, is not guaranteed or endorsed by the publisher.
